# Physical literacy in school aged children: a preliminary analysis relating health factors

**DOI:** 10.3389/fpubh.2025.1424027

**Published:** 2025-04-02

**Authors:** Noelia Mayordomo-Pinilla, Pedro Antonio Sánchez-Miguel, Carmen Galán-Arroyo, Antonio Castillo-Paredes, Jorge Rojo-Ramos

**Affiliations:** ^1^BioErgon Research Group, Faculty of Sports Sciences, University of Extremadura, Cáceres, Spain; ^2^Department of Didactics of Musical, Plastic and Body Expression, Faculty of Teaching Training, University of Extremadura, Cáceres, Spain; ^3^Grupo AFySE, Investigación en Actividad Física y Salud Escolar, Escuela de Pedagogía en Educación Física, Facultad de Educación, Universidad de Las Américas, Santiago de Chile, Chile; ^4^Promoting a Healthy Society Research Group (PHeSO), Faculty of Sport Sciences, University of Extremadura, Cáceres, Spain

**Keywords:** physical literacy, education, physical activity, age, sex, BMI, students, school location

## Abstract

**Introduction:**

Adolescents with high physical literacy (PL) have better health including cardiovascular fitness and reduced obesity. Improving these skills can lead to a healthier adulthood and reduce the risk of developing chronic diseases. However, physical activity (PA) levels are alarmingly low and PL has been proposed as a tool to improve them. This study aimed to investigate PL in school students [aged between 8 and 18 years (M = 13.08)] in southwestern Spain, explore sex differences and school location, and explore the correlations among PL, age, and Body Mass Index (BMI).

**Methods:**

The Mann-Whitney U test was applied to determine the differences in the PPLI-Q according to sex and school location variables and the Bonferroni correction. Spearman's Rho was also applied to determine the correlations between PL and BMI and age, determining the effect size using Hedge's g for sex and high school location. Results: The results showed significant differences in sex and high school location in favor of boys (M = 4.12, SD = 0.56) and rural settings (M = 4.11, SD = 0.52). The correlation between BMI and PL was significant, with an inverse and medium effect (BMI, *ρ* = −0.102, *p* = 0.006^*^).

**Discussion:**

These results suggest that females have a lower PL perception, and its correlation with BMI underscores the importance of intervention in improving their health. It is also important to intervene in the same way in older students and those with a higher BMI to improve their PL and increase their PA practices to establish healthier habits.

## 1 Introduction

According to the United Nations Educational, Scientific, and Cultural Organization (UNESCO), continuing professional development for teachers should be a key element of any national quality physical education (PE) strategy, and that quality PE should be an integral part of the school curriculum. In addition, UNESCO has emphasized that high-quality PE ought to be a fundamental part of school curricula since it serves as the foundation for adolescents' lifetime participation in physical activity (PA) and sports (1). In other words, a high-quality PE program aims to foster an interest in sports, regular PA, and an active, healthy lifestyle (1, 2). Furthermore, it was found that adolescents who participated in PE classes 1–2 days/week were 26% more likely to be sufficiently active, with somewhat greater chances for boys (30%) than girls [15%; (3)].

Following the international recommendations of the World Health Organization [WHO; (4, 5)], children and adolescents should engage in at least 60 min per day of moderate to vigorous PA, primarily aerobic, as well as bone-strengthening exercises, at least 3 days per week, for PA to be considered healthy. In addition to helping people maintain a healthy weight and level of fitness, engaging in PA has several physiological, psychological, and social advantages, such as enhancing cardiometabolic health, improving cognitive functioning, and lowering the risk of depression (4, 6, 7). However, according to a study by Guthold et al. (8), 81% of adolescents between the ages of 11 and 17 years are inactive, meaning that they do not satisfy the guidelines for physical exercise. Steene-Johannessen et al. (9) found that 71% of young people between the ages of 10 and 18 were not active. Specifically, in Spain, more than 80% of teenagers are inactive, according to Santos-Labrador (10), and nearly 90%, according to Castañeda-Vázquez (11). In the Spanish context, studies have shown a progressive decrease in the practice of physical activity from adolescence onwards, with children engaging in a greater volume and intensity of physical activity than adolescents. In terms of sex, adolescent girls practice less volume and intensity than young men. The data revealed that at least 55.4% of the children and adolescents did not meet international recommendations for physical activity (12).

Likewise, the phrase “physical literacy” (PL) has come to be used frequently in the context of PE by educators and other specialists involved in the growth of athletes (13) because it has become a crucial component of PA promotion (14). Cairney et al. (15) recognized PL as a process for developing and increasing PA levels in adolescents. The definition of PL is meant to aid PE teachers in establishing quality PE curricula, extracurricular activities, and health promotion programs designed to foster the development of PL among adolescents, since PL is the ultimate goal of PE (16). PL is not only implemented in educational settings where children and adolescents are involved but is also effective in work, family, health, and social settings where adults and seniors are also involved (17). PL is a complex and multifaceted concept (18) that refers to the motivation, self-assurance, physical skill, knowledge, and understanding that people acquire to maintain a healthy level of PA throughout their lives (17). PL is an evolving process in which the different components—physical competence, everyday behavior, knowledge and understanding, motivation, and confidence—interact holistically to promote lifetime involvement in and pleasure from PA (16, 18). PL is a unique type of intelligence associated with the ability to produce movement developed by engaging in PA. Nevertheless, PL is not the same as PA, but rather a vital precursor of PA (19).

PL presents a broader concept of PA that goes beyond ability and encompasses a wide range of activities beyond school-based PE or organized sporting events. PL promises more accurate representations of physical ability and PA for a larger population by utilizing pedagogies and adopting new ways of thinking, thus providing opportunities for everyone to become active and motivated participants (20). Adolescents have the right to high-quality PE that promotes PL and the development of the following key characteristics: a) physical self-esteem and self-confidence; b) participation motivation; c) how they interact with their environment, express themselves, and interact with others; and d) knowledge and understanding of how to maintain PA (17). Accordingly, teenagers gain self-assurance in basic motions, coordination, and control with regard to changing surroundings as a result of their improved PL. In addition, to communicate with others in a physical setting and take pleasure in learning new PAs, they can also exhibit verbal and non-verbal communication (16).

Therefore, PL is an important factor in the development of healthy habits in children and adolescents, constituting a tool for the prevention of risk factors such as childhood obesity, which currently expresses alarmingly high rates that are in turn associated with metabolic disorders in adulthood (21, 22). According to a systematic review of the blank spots in the literature on physical literacy and other systematic reviews on the subject (23), one of the major problems in the current state is the lack of studies on physical literacy levels and their determinants. This paper focuses on exploring students' sex, age, and rural location in terms of their perception of physical literacy. As for research comparing rural vs. urban environments, there are few studies with a clear conclusion; most point to a higher level of physical literacy in rural environments (24). However, most research comparing areas of residence looks at levels of physical practice rather than literacy. The scientific literature has thus far explored the influence of age on PL, finding direct relationships between this construct and healthy aging (25); however, other studies have found consistent correlations between age and PL, although more research is needed in adolescent population (26). Different studies have found differences in the perception of physical literacy between the sexes, with boys tending to have a higher level of literacy than girls (27). In addition, between environments, urban adolescents tend to have lower literacy levels (28). During adolescence, the volume and intensity of PA tends to decrease, resulting in a worsening of cardiorespiratory health and physical fitness in young people, especially in the female sex (29), which is associated with an increase in fat mass, worsening of body composition, and increased BMI (30). In this line related to adolescent health, different studies have stated that, although the lipid profile is not associated with physical literacy, it does correlate with the physical fitness of young people, such as a direct relationship with the level of physical activity and behaviors associated with better health (31). In addition, a systematic review and meta-analysis found a direct correlation between physical literacy and cardiorespiratory fitness (32), which is an important predictor of health at all stages of life, especially adolescence (21).

BMI is a measure of body mass based on a person's weight and height, and is used to classify individuals into categories such as underweight, normal weight, overweight, and obese. It is a widely used measure to define anthropometric height/weight characteristics in adults and children, and to detect weight categories that may lead to health problems (33). An increased BMI is a risk factor associated with health, as it correlates with a worse lipid profile and the development of cardiovascular diseases such as hypertension and diabetes (34), but is also associated with other unhealthy habits such as physical inactivity and poor nutrition (35). PL can prevent this decline in PA and improve adolescent health in addition to developing healthy habits that can be maintained in adulthood (36). In line with all the changes brought about by the shift in life habits from childhood to adolescence, PL is related to them. Research has revealed a correlation between BMI and this construct of inverse character, while it is also related to the level of PA practice (37). Furthermore, in the study of variables that may modify the acquisition of this construct, the results suggest that sex may influence PL (38). The study of different strategies that can be applied in educational and extracurricular centers to increase PE is of great importance, since an increase in PE can translate into an improvement in health and physical fitness (39), the establishment of healthy habits (2), and the development of prosocial behaviors (40). The importance of this study lies in identifying the factors that should be taken into account to improve it, as well as in studying which variables can influence its acquisition.

Spain's education system is divided into three primary segments. The first is infant education, which is mandatory for children aged three to six. The second is primary education, consisting of three cycles lasting 2 years each: the initial cycle spans 6 to 8 years, the second from 8 to 10 years, and the third from 10 to 12 years, all compulsory. The third segment is secondary education, comprising a 4-year block known as Compulsory Secondary Education (ESO), mandatory by nature, and another block called Baccalaureate, a 2-year non-compulsory cycle. PE is a compulsory subject in all grades, except in the second year of high school, where it is offered as an optional subject. The content taught in physical education varied depending on the educational stage of the student. During the primary stage, the focus is on the development of healthy life habits through physical activities, adopting behaviors that enhance physical, mental, and social health, in addition to adapting the elements of the body scheme, physical abilities applying decision processes appropriate to the internal logic, responding to the demands of daily life, and developing self-regulation processes within the framework of motor practice, with empathetic and inclusive attitudes regardless of ethnocultural, social, and gender differences (41, 42). In secondary education, the pedagogical objectives move toward the planning of PE and consider the responsibility in the decision-making of this planning, taking into account the inclusion of all participants. The acquisition of these competencies evolves during every educational stage but becomes sequentially complex (43).

After consulting the literature on this subject, it seems that the associations between PL and different sociodemographic variables such as school environment, which lacks specific research, or sex, age, and BMI, which have been published, although without absolute contributions, have not yet been established in a robust manner. In this sense, it seems logical to carry out research that contemplates associations with these variables. Therefore, this study aimed to determine the significant differences between sexes and school location, as well as to explore the possible correlations between BMI and the age of the sample in relation to physical literacy in Spanish adolescents.

## 2 Materials and methods

### 2.1 Participants

The method applied in this study uses a cross-sectional model. A non-probabilistic sampling method based on convenience sampling was applied to calculate the appropriate sample size for this study (44). Table 1 presents the sociodemographic characteristics of the sample, being fairly balanced in terms of sex, since 50.6% (*N* = 366) were boys and 49.4% (*N* = 357) girls. The location of the school had similar characteristics: 48% attended a rural educational school, and 52% attended an urban area. The criterion for determining whether an institute is located in a rural or urban setting is the number of inhabitants of the locality: if the locality has < 20,000 inhabitants, it is considered rural; on the other hand, if the number of inhabitants is above this figure, it is urban. This criterion can be consulted at the Diputación de Cáceres (https://www.dip-caceres.es/). The mean age of the sample was 13.08 years with a weight of 57.9 kg and an average height of 1.57 meters, with a mean BMI of 22.70 kg/m^2^.

**Table 1:** Sample characterization (*N* = 723).

**Variable**	**Categories**	**N**	**%**
Sex	Boys	366	50.6
	Girls	357	49.4
Grade	2^nd^ Grade	2	0.3
	3^rd^ Grade	6	0.8
	4^th^ Grade	47	6.5
	5^th^ Grade	93	12.9
	6^th^ Grade	119	16.5
	1^st^ CSE (Compulsory Secondary Education)	161	22.3
	2^nd^ E.S.O.	141	19.5
	3^rd^ E.S.O.	83	11.5
	4^th^E.S.O.	56	7.7
	1^st^ Baccalaureate	13	1.8
	2^nd^ Baccalaureate	2	0.3
School location	Rural	347	48
	Urban	376	52
**Variable**		**M**	**SD**
Age		13.08	1.78
Weight		57.90	15.13
Height		1.57	0.14
BMI		22.70	2.39

N, number; %, percentage; SD, standard deviation; M, Mean.

To participate in the study, participants had to meet a series of requirements and criteria: 1. To obtain informed consent from their parents as minors, 2. To attend PE in a public school located in Extremadura (aged 8–18 years).

All the data were collected anonymously and kept private. The study was performed according to the guidelines of the Declaration of Helsinki and was approved by the Bioethics and Biosafety Committee of the University of Extremadura (protocol code: 186/2021).

### 2.2 Procedure

To contact the schools whose students would participate in the research, we accessed the directory of public schools in Extremadura available at the Department of Education and Employment of the Regional Government of Extremadura, obtaining contact information of the institutions that teach secondary education (8–18 years of age) in this autonomous community.

Once the contacts of the schools had been compiled, we sent an e-mail to the PE teachers with information on the study, its design, intervention, and objectives; a model of the instrument to be applied; and informed consent to be signed by the parents of the students. In the event that the teacher agreed and decided to participate in the study, he/she would inform the researcher to subsequently set an appointment in which the research team would go to the school to implement the questionnaires to the PE students after collecting the informed consent duly signed by the students' parents.

The questionnaire was administered on the day of the research implementation by two members of the investigation group utilizing a tablet containing the link to Google Forms, where the questionnaires were located. After all participants who signed the consent form read the items aloud, the items were explicated to ensure accurate comprehension. Electronic questionnaires are more cost-effective and facilitate data collection by streamlining the storage of responses in a unified database. The estimated response time for the questionnaires did not exceed 10 min, and data were collected between September and December 2022.

### 2.3 Instruments

Although objective measures (such as accelerometers, physical tests, and pedometers) and subjective measures (such as questionnaires) both contribute to a better understanding of PL (45), adding objective measures requires a substantial amount of money, space, and testing time. Therefore, self-report surveys are simpler to use when it is not possible to include additional objective measurements (46). In this sense, the following were applied:

#### 2.3.1 Sociodemographic data

A questionnaire was designed to obtain information on the characteristics of the sample, with six questions on sex, age, height, weight, grade, and school location.

#### 2.3.2 Perceived physical literacy instrument (PPLI) for adolescents

In this sense, the PPLI, which includes nine items covering three different categories, was one of the self-reported PL instruments for teenagers that Sum et al. (47) validated. This was followed by further validation for adolescents (47), which contained nine distinct questions from the original instrument (48), divided into three different domains: “knowledge and understanding,” “self-expression and communication with others, and ”sense of self and self-confidence“ with three items, respectively. However, because this evaluation instrument demonstrated good validity in a systematic review that included the assessment tools already available (49), López-Gil et al. (16) chose to adapt and validate it in Spanish. Finally, the Spanish Perceived Literacy Instrument for Adolescents (S-PPLI), whose nine items are graded on a 5-point Likert scale from 1 (strongly disagree) to 5 (strongly agree), was employed as the tool to analyze PL. The authors reported satisfactory reliability for this scale with a Cronbach's alpha coefficient of 0.87. In addition, confirmatory factor analysis (CFA) showed that the responses adequately conformed to the three-factor structure (χ^2^ = 52.260, df = 24, *p* < 0.001, CFI = 0.976, RMSEA = 0.057, SRMR = 0.031).

### 2.4 Statistically analysis

First, the distribution of the data was examined to determine whether the assumption of normality was met to determine the type of statistical test to be used. The Kolmogorov-Smirnov test was used for this purpose. The result of this test showed that this assumption was not met (*p* < 0.005). Therefore, non-parametric statistical tests were performed.

The Mann-Whitney U test was used to analyze the differences in the scores of each item of the PPLI-Q for adolescents and the physical alpha-sensitization construct as a function of sex or demographic location of the participants according to their age, dividing the sample into two groups: 8–12 and 13–18 years old. Bonferroni correction was applied for the multiple comparisons performed in the analyses of each item of the instrument according to sex and demographic location, so that a significance value of *p* < 0.005 was established. For the rest of the analyses, a significance value of *p* < 0.05 was established.

For the interpretation of BMI results, percentiles were established with three cutoff points at 25, 50, and 75%. These values were adjusted for age and sex according to Cole [2000, 2007; (50, 51)].

Spearman's rho test was used to determine the degree of relationship between PL and age or BMI. For the interpretation of this statistic, we took into account the ranges established by Mondragón-Barrera (52) who determined that coefficients between 0.01 and 0.10 determined the existence of a low correlation, values between 0.11 and 0.50 implied a medium degree of correlation, from 0.51 to 0.75 a strong correlation, from 0.76 to 0.90 a high correlation and above 0.91 the correlation was perfect.

Finally, Cronbach's Alpha and McDonald's omega coefficients were used to evaluate the reliability of the psychometric scales, based on their internal consistency. To interpret the values reported, those established by Nunnally and Berstein (53) were chosen, which indicated that values below 0.70 would correspond to low reliability, values between 0.71 and 0.90 would correspond to satisfactory reliability, and values above 0.91 would correspond to excellent reliability. The maximum likelihood model (Omega ML) was used to calculate McDonald's Omega coefficient. Both Cronbach's α and McDonald's ω coefficients were used to assess the reliability of the instruments with the aim of making a more complete and accurate assessment of internal consistency. The inclusion of both coefficients in the research responds to the need to address the inherent methodological limitations of each of the measures and to provide more solid reliability information, taking advantage of the specific strengths of each.

The data are presented as numbers and percentages for the sociodemographic variables and as mean (M) and standard deviation (SD) for the scores obtained in each of the items of the PPLI-Q for adolescents. The Statical Package of Social Science was used for data analysis in version 27 for the MAC.

## 3 Results

Table 2 shows the results obtained from the analysis of the items of the PPLI-Q questionnaire according to sex divided into two age groups: 8 to 12 years old and 13 to 18 years old. No significant differences were found in the first age group; however, in the older group, significant differences were found in most items, with boys obtaining a higher score in all cases.

**Table 2:** Scores and differences according to sex and age group were obtained for the PPLI-Q items.

**Item**		**Global scores**		**8–12 years (*****N*** = **267)**					**13–18 years (*****N*** = **456)**				
		**Sex**			**Sex**					**Sex**			
		**Boys**	**Girls**		**Boys**	**Girls**	***p***	**g**		**Boys**	**Girls**	***p***	**g**
	**M (SD)**	**M (SD)**	**M (SD)**	**M (SD)**	**M (SD)**	**M (SD)**			**M (SD)**	**M (SD)**	**M (SD)**		
1. I am physically fit, in accordance with my age.	4.21 (0.98)	4.26 (1.03)	4.16 (0.93)	4.27 (1.03)	4.22 (1.13)	4.34 (0.91)	0.638	0.116	4.17 (0.95)	4.28 (0.97)	4.07 (0.93)	0.002^*^	0.232
2. I have a positive attitude and interest in sports.	4.28 (1.04)	4.42 (0.97)	4.15 (1.08)	4.45 (0.96)	4.39 (1.05)	4.53 (0.83)	0.569	0.146	4.19 (1.07)	4.43 (0.92)	3.97 (1.15)	< 0.001^*^	0.439
3. I appreciate myself or others doing sports	4.12 (0.98)	4.26 (0.97)	3.97 (0.97)	4.23 (1.06)	4.23 (1.07)	4.23 (1.06)	0.987	0.000	4.05 (0.93)	4.29 (0.91)	3.85 (0.91)	< 0.001^*^	0.484
4. I possess self-management skills for fitness	3.58 (1.19)	3.78 (1.21)	3.37 (1.14)	3.63 (1.26)	3.72 (1.32)	3.51 (1.19)	0.089	0.166	3.55 (1.15)	3.83 (1.29)	3.31 (1.12)	< 0.001^*^	0.432
5. I possess self-evaluation skills for health	3.78 (1.08)	3.86 (1.09)	3.70 (1.06)	3.85 (1.13)	3.93 (1.45)	3.74 (1.13)	0.128	0.144	3.74 (1.04)	3.8 (1.06)	3.68 (1.03)	0.121	0.115
6. I have strong social skills	3.95 (1.01)	3.98 (1.03)	3.93 (1.00)	3.95 (1.02)	3.93 (1.08)	3.97 (0.96)	0.950	0.038	3.96 (1.01)	4.02 (1.00)	3.9 (1.02)	0.216	0.119
7. I am confident in wild/natural survival	3.67 (1.10)	3.86 (1.12)	3.47 (1.05)	3.85 (1.15)	3.95 (1.12)	3.73 (1.42)	0.151	0.175	3.56 (1.06)	3.8 (1.12)	3.34 (0.96)	< 0.001^*^	0.443
8. I am capable in handling problems and difficulties during sports	3.87 (0.94)	3.90 (0.95)	3.83 (0.94)	3.72 (1.03)	3.76 (1.09)	3.66 (0.97)	0.295	0.096	3.96 (0.88)	4.00 (0.84)	3.91 (0.92)	0.383	0.102
9. I am aware of the benefits of sports related to health	4.55 (0.83)	4.61 (0.83)	4.50 (0.82)	4.51 (0.90)	4.51 (0.99)	4.51 (0.80)	0.237	0.000	4.58 (0.78)	4.68 (0.70)	4.49 (0.84)	0.001^*^	0.244
**Perceived physical literacy**	4.00 (0.59)	4.10 (0.61)	3.89 (0.54)	4.05 (0.61)	4.06 (0.70)	4.02 (0.49)	0.074	0.000	3.97 (0.57)	4.12 (0.56)	3.83 (0.56)	< 0.001^*^	0.518

*p* is significant < 0.05^*^. M, mean value; SD, Standard deviation. Each score is based on a Likert scale (1–5): 1 (strongly disagree), 2 (disagree), 3 (indifferent), 4 (agree), and 5 (strongly agree).

Table 3 shows the data obtained from the analysis according to school location divided into the same age ranges as in the previous analysis. In this case, in the 8–12 age group, significant differences were obtained in items 1 and 7 only, with students from rural areas obtaining higher scores. In the case of the 13–18 age group, a large majority of the items expressed significant differences, with the rural environment scoring the highest.

**Table 3:** Scores and differences according to school location and age group were obtained for the PPLI-Q items.

**Item**	**Global sores**		**8–12 years (*****N*** = **267)**				**13–18 years (*****N*** = **456)**			
	**School location**		**School location**				**School location**			
	**Rural**	**Urban**	**Rural**	**Urban**	***p***	***g***	**Rural**	**Urban**	***p***	***g***
	**M (SD)**	**M (SD)**	**M (SD)**	**M (SD)**			**M (SD)**	**M (SD)**		
1. I am physically fit, in accordance with my age.	4.37 (0.91)	4.06 (1.02)	4.42 (0.91)	4.13 (1.14)	0.035^*^	0.281	4.34 (0.92)	4.02 (0.96)	< 0.001	0.340
2. I have a positive attitude and interest in sports.	4.43 (0.93)	4.15 (1.11)	4.52 (0.87)	4.38 (1.05)	0.483	0.145	4.38 (0.98)	4.02 (1.13)	< 0.001	0.340
3. I appreciate myself or others doing sports	4.23 (0.90)	4.01 (1.03)	4.3 (1.00)	4.16 (1.12)	0.297	0.132	4.19 (0.85)	3.93 (0.99)	0.006	0.280
4. I possess self-management skills for fitness	3.62 (1.21)	3.54 (1.17)	3.71 (1.23)	3.54 (1.31)	0.352	0.130	3.57 (1.21)	3.54 (1.10)	0.584	0.333
5. I possess self-evaluation skills for health	3.80 (1.08)	3.76 (1.08)	3.93 (1.11)	3.77 (1.17)	0.25	1.140	3.72 (1.06)	3.75 (1.04)	0.799	0.030
6. I have strong social skills	4.11 (0.92)	3.81 (1.07)	4.09 (0.87)	3.8 (1.15)	0.071	0.285	4.12 (0.92)	3.82 (1.04)	0.001	0.304
7. I am confident in wild/natural survival	3.88 (1.06)	3.47 (1.10)	4.01 (1.08)	3.7 (1.21)	0.04^*^	0.270	3.81 (1.06)	3.34 (1.03)	< 0.001	0.450
8. I am capable in handling problems and difficulties during sports	4.01 (0.96)	3.74 (0.91)	3.75 (1.07)	3.68 (1.01)	0.401	0.067	4.17 (0.86)	3.77 (0.86)	< 0.001	0.465
9. I am aware of the benefits of sports related to health	4.65 (0.68)	4.46 (0.93)	4.58 (0.8)	4.44 (1.00)	0.347	0.155	4.70 (0.60)	4.48 (0.90)	0.009	0.283
**Perceived physical literacy**	4.12 (0.50)	3.88 (0.63)	4.14 (0.49)	3.95 (0.71)	0.111	0.312	4.11 (0.52)	3.85 (0.60)	< 0.001	0.460

The ^*^ means statistical significance (*p* < 0.05).

The following table (Table 4) shows the results of the correlation analysis between the questionnaire items and the variables age and BMI. Significant correlations were identified between age and items 1, 2, and 7, with inverse associations and a medium degree of correlation, explaining that the higher the age, the lower the score obtained for these items, and therefore, the worse the perception of PL. Similarly, item 8 obtained significant differences, although the correlation in this case was positive and direct with a medium degree of correlation, revealing that the higher the age, the higher the score. Regarding BMI, significant associations were found for Item 2 and the general score of the questionnaire.

**Table 4:** Correlation between PPLI-Q scores for adolescents' variables and age and BMI.

**Items**	**Age** ***ρ** **(p)***					**BMI** ***ρ** **(p)***				
	**Overall correlation**	**Rural**	**Urban**	**Boys**	**Girls**	**Overall correlation**	**Rural**	**Urban**	**Boys**	**Girls**
1. I am physically fit, in accordance with my age.	−0.126 (0.001)	−0.999 (0.066)	−0.134 (0.009)	−0.028 (0.595	−0.232 (< 0.001)	−0.039 (0.290)	−0.066 (0.221)	−0.058 (0.261)	−0.040 (0.441)	−0.003 (0.952)
2. I have a positive attitude and interest in sports.	−0.141 (0.001)	−0.050 (0.354)	−0.196 (< 0.001)	−0.006 (0.902)	−0.275 (< 0.001)	−0.120 (0.001)	−0.060 (0.265)	0.197 (< 0.001)	−0.087 (0.095)	−0.100 (0.058)
3. I appreciate myself or others doing sports	−0.090 (0.016)	−0.049 (0.363)	−0.114 (0.027)	0.036 (0.489)	−0.222 (< 0.001)	−0.068 (0.066)	−0.062 (0.252)	−0.107 (0.037)	−0.017 (0.739)	−0.044 (0.407)
4. I possess self-management skills for fitness	−0.035 (0.347)	−0.015 (0.776)	−0.052 (0.318)	−0.009 (0.859)	−0.055 (0.303)	−0.093 (0.012)	−0.041 (0.444)	– 0.147 (0.004)	−0.036 (0.492)	−0.098 (0.065)
5. I possess self-evaluation skills for health	−0.074 (0.048)	−0.098 (0.068)	−0.054 (0.299)	−0.061 (0.246)	−0.084 (0.114)	−0.066 (0.075)	−0.110 (0.040)	−0.029 (0.581)	−0.052 (0.323)	−0.046 (0.383)
6. I have strong social skills	−0.006 (0.882)	0.024 (0.658)	−0.008 (0.872)	0.003 (0.953)	−0.010 (0.855)	−0.044 (0.233)	−0.031 (0.569)	−0.073 (0.159)	−0.049 (0.353)	−0.012 (0.824)
7. I am confident in wild/natural survival	−0.152 (< 0.001)	−0.101 (0.059)	−0.163 (0.001)	−0.063 (0.228)	−0.224 (< 0.001)	−0.090 (0.010)	−0.145 (0.007)	−0.089 (0.086)	– 0.053 (0.316)	−0.072 (0.172)
8. I am capable in handling problems and difficulties during sports	0.141 (< 0.001)	0.210 (< 0.001)	0.109 (0.034)	0.118 (0.024)	0.164 (0.002)	0.041 (0.266)	0.049 (0.361)	0.016 (0.752)	0.041 (0.439)	0.062 (0.244)
9. I am aware of the benefits of sports related to health	0.057 (0.123)	0.096 (0.073)	0.039 (0.456)	0.063 (0.230)	0.070 (0.188)	−0.021 (0.577)	−0.007 (0.903)	−0.052 (0.310	−0.017 (0.745)	0.023 (0.670)
Perceived physical literacy	−0.072 (0.052)	0.014 (0.797)	−0.107 (0.038)	0.006 (0.902)	−0.158 (0.003)	−0.102 (0.006)	−0.097 (0.071)	−0.146 (0.005)	−0.076 (0.144)	−0.058 (0.271)

Each score obtained on the PPLI-Q for adolescents is based on a Likert scale (1–5).

The reliability of the instrument was calculated based on its internal consistency using Cronbach's alpha (α = 0.750) and McDonald's omega (ω = 0.747). These values can be considered satisfactory according to Nunnally and Bernstein (53).

## 4 Discussion

The work carried out in this article focused on exploring the behavior of sex and school location variables in relation to PL, in addition to studying the correlations between PL and age and BMI in secondary school students in Extremadura using the PPLI-Q for Adolescents questionnaire. In general, significant differences were found between the sexes and school locations. Similarly, for both variables, differences were found in several questionnaire items. In addition, no significant correlations were found between PL and BMI or age; however, moderate correlations were observed for certain items.

In the analysis of the questionnaire items differentiated by sex, the scores obtained by both sexes were significantly high, generally reaching 4 out of 5 points and sometimes more than 4.5 points on this scale. Other studies have established that the levels of PL are generally high, being above 3 on a 5-point scale (54, 55). They also suggest that age influences these levels, since students belonging to higher grades obtain higher values (55) as in the results of this work. In all age groups, generally high scores were obtained, although in older students, the difference between sexes was much more notable than in younger ones. It is difficult to compare these results with the scientific literature, as there is no research that divides this assumption into similar ages. Regarding sex differences, boys showed a higher knowledge of PL in all items and at a general level than girls in all age groups, although no significant differences were found in youngest students, partially accepting hypothesis number 1. Studies carried out in this field of research reveal a relationship between the level of PA and PL, explaining that individuals who perform a greater volume and intensity of PA have, in general, greater PL, since PA has a positive influence on this variable (30, 56, 57). In line with this statement, despite the fact that recent trends reveal that PA practice is becoming more equitable, boys continue to perform more PA at a higher intensity than girls in Spain (58), therefore, the results obtained may be due to this difference in the level of PA practice. Continuing with the results obtained in this study, other studies have reported very similar effects in terms of sex differences, with boys scoring higher than girls (56, 59). However, not all studies support these results. Different studies have found no significant sex differences, although male students scored higher (54, 60). The items without statistical significance belong to the dimension ”self-expression and communication with others,” suggesting that sex is not an influential factor in the expression of PL as it relates to the sex of secondary students. These results are not in agreement with those of other studies that have found significant correlations (61). Sex differences in PA play a crucial role in the acquisition of PL. In this sense, the practice of PA alone in children is not sufficient to establish healthy habits (62). The difference in this practice and in the development of social skills through PA is perhaps the reason why this dimension did not show significant differences. However, further research is required in this area. Taking the latter research as a guide, the effect size of sex on PL is generally low or null for certain items. Therefore, these results should be cautiously interpreted.

The scientific literature related to the study of the environmental characteristics of students establishes the demographic area as an element of high relevance (24). This location may influence the PL perceived by students, stating that those belonging to rural areas may have a lower level of this construct because of a lack of resources. However, the results obtained in this study coincide on the association side, suggesting that the location of schools may influence students' PL. Contrarywise, the scores of rural students were higher, accepting H_2_. Knowing the concept of PL and its close and reciprocal relationship with PA (57), which may be due to greater PA outside the school environment, since, not being urban centers, they have more and larger spaces available for motor development and PL, specifically in Spanish students (28). Özdiren and his collaborators found a higher rate of PA in rural environments than in urban environments (63), which, linked to the association of greater PL the higher the PA, may answer the results obtained in this study. In this matter, there are more contradictions in the scientific literature since multiple studies have revealed significant differences in favor of urban environments over rural ones (60, 64). In contrast, other studies have shown results that coincide with those of this research, with the rural environment obtaining the highest score on PL (24, 65) related to the above, y. However, further research in this field is required for more complete and fruitful discussion.

Continuing with the results obtained in the study of the correlations between BMI and age related to PL, significant associations were found in four variables of age with mean and inverse characteristics, partially accepting hypothesis 3 and 4. In the study on the association between BMI and PL, previous studies showed that those with a healthy weight showed a higher correlation with this variable, being an inverse correlation in general (66), in the same line as that found in this work. The authors suggested that a higher level of physical fitness corresponding to a higher level of physical activity, is due to a higher level of physical activity (67). These items revealed that interest in sports and self-confidence in the development of sports activities decrease as the individual gets older. However, no significant association was observed in the overall results. In relation to these results, scientific literature provides similar results, showing that adolescents have lower PL than children (54, 65), accompanied by a decrease in self-esteem and self-concept (68). PL and, therefore, PA are closely related to these two concepts; the implementation of PA programs and improved physical fitness can improve self-esteem in this population (69).

Additionally, inverse and mean correlations were found in multiple items of the two variables, with the exception of the section “I am capable in handling problems and difficulties during sports,” which expresses positive correlations. In this sense, the analysis revealed that, in general, younger students are more physically literate than their older peers in terms of age. In line with these findings, research has found an association between knowledge about this variable and the practice of PA, and as previously described, as age increases and adolescence progresses, less PA is performed (57, 70). Other studies that focused on the exploration of age in this area also did not find correlations or significant age-related differences (59, 60). In contrast, other studies have found significant positive correlations, revealing that the older the age, the greater the knowledge about PL they possess (54, 71). Conversely, inverse associations of average characteristics were also found for BMI. In this case, scientific literature reports similar results (66, 72, 73). In a study conducted by Mendoza-Muñoz et al. in 2021, those who were overweight had worse PL than their normal-weight peers, claiming that the latter group performed more daily PA than those with a higher BMI (74).

After learning about these results, different points were identified: PL is interconnected with the level of PA performed; therefore, advocating for a healthier and more active future for students, educational interventions that improve this knowledge are needed to achieve holistic results of PA interventions in this aspect, improving PA levels through better PL. Furthermore, these results indicated profiles where these indicators were lower; girls scored lower on this questionnaire, indicating a lower level of PA. As for age and BMI, it is important to act on older, overweight, or obese adolescents, as they are the lowest scorers and may benefit from prosocial effects acquired through PA and higher PL. A comprehensive educational intervention for these more vulnerable and lower-scoring profiles may improve physical inactivity rates by increasing knowledge of PL, specifically in girls, older students, and overweight students, making PE an ideal medium to target these aspects through workshops or interventions. In the context of strategic planning, it is imperative to increase, through institutional initiatives, the availability of urban spaces where youth can engage in physical activities.

Among the strengths of this work is the novelty in the study of PL in its demographic characteristics related to the school environment, which is one of the first investigations carried out in this aspect. This study had several limitations that should be considered in future research. First, it is important to note that all participants were exclusively drawn from an autonomous community in Extremadura, Spain. While this allowed for a focused examination within this specific cultural context, it also raised concerns regarding the broader generalizability of the findings to populations with distinct cultural backgrounds. However, the design of this study did not consider the physical activities performed by students during out-of-school hours.

Furthermore, the study's cross-sectional and non-probabilistic design imposes constraints on the ability to infer the causation between PL and body composition. The temporal nature of cross-sectional studies makes it challenging to establish definitive causal relationships. Additionally, e-questionnaires were self-administered so children might not respond accordingly. Therefore, future research employing longitudinal approaches may offer valuable insights into the dynamic interplay between PL and body composition. In addition, electronic questionnaires were used, which despite their advantages, have certain disadvantages that should be considered.

The study of PL in rural and urban environments is a line of study that is little exploited and has few quality results available; therefore, in order to increase scientific knowledge about the behavior of PL, it is interesting to increase the study of this variable. There are multiple contradictory results in the scientific literature on the behavior of PL across age groups, making this an interesting line of research. It would be interesting to extend the study population to other regions in Spain to reduce the cultural limitations of this study.

## 5 Conclusion

In conclusion, this study highlights factors influencing physical literacy. The observed sex disparity (girls score lower on the PL questionnaire) requires sex-sensitive interventions to ensure equal opportunities. In addition, it is essential to address differences related to age and BMI, especially in the case of older, overweight or obese adolescents, who have the lowest scores. Tailor-made educational interventions targeting these vulnerable groups can significantly improve PL and consequently reduce rates of physical inactivity. Furthermore, results comparing physical literacy levels between rural and urban areas showed a disparity in favor of rural areas, which may be due to the restriction of play areas in cities, in addition to the difference in perceived safety, where parents perceive greater risk in cities than in towns. It would be interesting to extend the theoretical framework of the study of the differences between urban and rural areas, as there is little literature on the subject. In terms of lines of action, it seems necessary to increase the number of spaces in cities where young people can engage in physical activity. A comprehensive and inclusive educational approach is essential to foster a high PL generation, which contributes to both individual wellbeing and the wider health goals of society.

## Data Availability

The raw data supporting the conclusions of this article will be made available by the authors, without undue reservation.
